# Demonstration of Insect Vector-Mediated Transfer of a Betasatellite between Two Helper Viruses

**DOI:** 10.3390/v16091420

**Published:** 2024-09-05

**Authors:** Noun Fouad, Martine Granier, Stéphane Blanc, Gaël Thébaud, Cica Urbino

**Affiliations:** PHIM Plant Health Institute, CIRAD, INRAE, Univ Montpellier, Institut Agro, IRD, 34398 Montpellier Cedex 5, France

**Keywords:** *B. tabaci*, emerging disease, epidemiology, *Geminiviridae*, *Begomovirus coheni*, *Begomovirus gossypigeziraense*, *Betasatellite gossypigeziraense*, host range, risk, transmission

## Abstract

Begomoviruses, transmitted by the whitefly *Bemisia tabaci*, pose significant threats to global agriculture due to their severe impact on various crops. Among the satellite molecules associated with begomoviruses, betasatellites play a crucial role in enhancing disease severity and yield losses. The spread and association of these molecules with helper viruses in host plants are thus matters of concern. Here, we focus on the propagation of betasatellites and, more specifically, on their transfer between different helper viruses and hosts through vector transmission. Our results show that the cotton leaf curl Gezira betasatellite (CLCuGeB), initially acquired with its helper virus cotton leaf curl Gezira virus (CLCuGeV) from an okra plant, can be transmitted and assisted by a different helper virus, tomato yellow leaf curl virus (TYLCV), in a different host plant (tomato plant). The new association can be formed whether TYLCV and CLCuGeB encounter each other in a host plant previously infected with TYLCV or in whiteflies having acquired the different components separately. Our findings reveal two pathways by which betasatellites can be transferred between helper viruses and host plants and highlight the ability of betasatellites to spread in begomovirus-infected environments.

## 1. Introduction

Begomoviruses (Family *Geminiviridae*) are single-stranded DNA viruses causing important damage to different crops [1]. They are transmitted in a persistent circulative manner by whiteflies of the *Bemisia tabaci* complex [2,3,4], which are polyphagous insects present all over the world [5,6]. Due to this invasive vector and the difficulty in managing viral infections in intensive crop production, begomoviruses have expanded their geographic range in recent decades and are now considered as the main group of emerging plant viruses with a global impact [5,7]. They cause severe symptoms leading to substantial yield losses for various crops of the families *Solanaceae* (e.g., tomato, pepper, eggplant), *Cucurbitaceae* (e.g., squash, zucchini, watermelon), or *Malvaceae* (e.g., cotton, okra, hibiscus) in tropical and subtropical regions [5].

As for many plant viruses, begomoviruses interact with satellite molecules, which can affect virulence. Begomoviruses can be associated with three types of single-stranded DNA satellite molecules: alphasatellites (family *Alphasatellitidae*, subfamily *Geminialphasatellitinae*), betasatellites, and deltasatellites (family *Tolecusatellitidae*) [8,9,10]. Unlike alphasatellites and deltasatellites, which generally do not increase viral symptoms [9,10,11,12,13], betasatellites enhance the severity of begomovirus diseases and thus cause higher yield losses in crops [13,14]. In the 2000s, betasatellites were detected in association with begomovirus species in Asia and Africa. Their presence was shown to be, in some cases, essential for the success of host infection by the virus or the expression of typical disease symptoms [8,15,16,17]. These molecules do not code any replication or capsid protein and therefore depend on helper begomoviruses for their whole life cycle: replication, encapsidation in the helper viral coat protein, within-plant movement, and vector-mediated transmission. As the coat protein of begomoviruses is the determinant of vector transmission [18,19], they are transmitted in a circulative manner like their helper virus. Betasatellite genomes encode a single protein named βC1, which acts as a pathogenicity determinant enhancing the symptoms caused by begomoviruses [20,21,22], most likely through inhibition of RNA silencing [13,23,24]. Betasatellites can also enhance the replication and accumulation of begomoviruses in plants [8,16,25], which in turn may increase the efficiency of transmission by whiteflies and ultimately the spread of diseases [26].

The threats posed by betasatellites are amplified by their promiscuous way of life. Indeed, a betasatellite may be efficiently replicated by begomoviruses from distinct species [27], including viruses that have not coevolved with these betasatellites, and even viruses that had never been reported in association with a betasatellite [27,28,29,30]. This illustrates that betasatellites are a threat to begomovirus-infected crops, even if introduced without the original helper virus [13,31].

Several lines of evidence suggest that betasatellites spread beyond their native regions in new host plants or in association with different viruses. Cotton leaf curl Gezira betasatellite (CLCuGeB; *Betasatellite gossypigeziraense*) was first reported in Sudan in a cotton plant infected with the helper virus cotton leaf curl Gezira virus (CLCuGeV; *Begomovirus gossypigeziraense*) [32]. It was then detected with the same helper virus in okra in South Asia [33,34], Saudi Arabia [35], Jordan [36] and several African countries, including Sudan, Mali, Niger, and Burkina Faso [37,38,39,40]. CLCuGeB is necessary for the success of infection or symptom expression of CLCuGeV in okra plants [8]. However, CLCuGeB was also reported in plants other than okra or cotton, associated with begomovirus species other than CLCuGeV. It was reported in tomato plants infected with tomato yellow leaf curl Mali virus (*Begomovirus solanumflavusmaliense*) in Mali [28], tomato yellow leaf curl virus (TYLCV; *Begomovirus coheni*) and tomato yellow leaf curl Sardinia virus (*Begomovirus solanumflavusardiniaense*) in Jordan [41], TYLCV in Israel [42] and Iraq [43], or with squash leaf curl virus (*Begomovirus cucurbitapeponis*) and watermelon chlorotic stunt virus (*Begomovirus citrulli*) in the weed *Sinapis arvensis* in Jordan [44]. Other pieces of evidence show the possibility for betasatellites to change host plants, helper viruses, and even vectors. For example, CLCuGeB was detected with a different virus than CLCuGeV in whiteflies in Israel [45]. Additionally, associations have been reported between betasatellites and members of the genus *Mastrevirus* (usually transmitted by leafhoppers), such as wheat dwarf India virus (*Mastrevirus hordei*) in wheat [46] and chickpea chlorotic dwarf virus (*Mastrevirus cicerparvi*) in spinach [47]. Moreover, it was previously reported that an infectious clone of betasatellite can be assisted by different helper viruses [27,48], suggesting that betasatellite genome variations observed among field sequences do not determine the association with a given helper virus.

Although all these observations indicate that betasatellites are prone to moving from a helper virus to a different one and between host plants, the underlying processes have never been documented experimentally. It is thought that virions containing betasatellite DNA are acquired together with virions containing the helper virus DNA when whitefly vectors feed on coinfected plants; therefore, the transfer of a betasatellite between helper viruses may occur during transmission, following the encounter of the betasatellite and the different helper viruses in the whiteflies or in the plant. Two mutually non-exclusive hypothetical scenarios leading to such a transfer have been tested in this study. The first is the acquisition of the original virus–betasatellite association, followed by transmission of the sole betasatellite in plants that are already infected with a different helper begomovirus. The second is the infection of healthy host plants with new virus–betasatellite associations by whiteflies having acquired a betasatellite and two different helper viruses from one or several source plants.

More specifically, using one of the most widespread betasatellites, CLCuGeB, and taking advantage of the host plant specificity of different helper viruses (okra for CLCuGeV, tomato for TYLCV), we aimed to assess two possible scenarios of whitefly-mediated transfer of a betasatellite between two helper viruses: (i) the acquisition of a betasatellite (CLCuGeB) and its original helper virus (CLCuGeV), followed by inoculation into TYLCV-infected tomato plants which the original helper virus cannot infect and (ii) the acquisition of CLCuGeB and the two helper viruses (CLCuGeV and TYLCV) followed by inoculation into the respective host plant of each helper virus.

## 2. Material and Methods

### 2.1. Viruses and Betasatellite

Agroinfectious clones of CLCuGeV, CLCuGeB, and the Israel strain of TYLCV were used in this study. TYLCV [RE:STG4:04] (accession number AM409201) was isolated from a tomato plant (*Solanum lycopersicum*) sampled in Réunion Island [49]. CLCuGeV [BF:Pô:Ok1:08] and CLCuGeB [BF:Kap:Ok1-2:08] (accession numbers FN554531 and FN554575, respectively) were isolated from an okra plant (*Abelmoschus esculentus*) sampled in Burkina Faso [39]. The construction of agroinfectious clones was previously described for TYLCV [50] and CLCuGeB [51]. Regarding CLCuGeV, full-length genomes were released from the pGEM-T Easy vector (Promega, Madison, WI, USA) by digestion with the restriction enzyme PstI and ligated as a tandem repeat into the corresponding restriction site of the binary vector pCAMBIA 2300 (accession number AF234315). *Agrobacterium tumefaciens* strain C58 (pMP90) [52,53] was transformed with the recombinant plasmids by electroporation.

### 2.2. Plant Material, Virus, and Betasatellite Inoculation

We agroinfiltrated the cotyledons of 29 14-day-old tomato plants (*Solanum lycopersicum*) of the variety ‘Monalbo’ with the agroinfectious TYLCV clone preparation as previously described [50]. In addition, 43 15-day-old okra plants (*Abelmoschus esculentus*) were inoculated with CLCuGeV and CLCuGeB using the biolistic method that comprises the following steps: tandem constructs of CLCuGeV and CLCuGeB were released from the recombinant plasmid pCAMBIA 2300 by digestion with SphI. To maximize the amount of viral DNA, the tandem construct of each genome was amplified using a GoTaq Long PCR Master Mix kit (Promega, Madison, WI, USA) and universal M13 primer sequences within the M13 bacteriophage-derived cloning vector. Each PCR was performed in a final volume of 10 µL containing 5 µL of GoTaq Long Buffer 2X, primer mix (M13-8436-F and M13-8333-R; Table 1), 2 µL of RNAse-free water, and 1 µL of tandem constructs. The PCR was run with the following conditions: initial denaturation at 95 °C for 2 min, followed by 30 cycles consisting each of 30 s denaturation at 94 °C, annealing and extension conditions as described in Table 1, and a 10 min final extension at 72 °C. Following gel purification, equal concentration of amplicons and tandems were mixed in 50 µL to reach a final concentration of 2 µg/µL for each genome. A mixture of 25 mg of 1 µm gold microbeads and 100 µL of 0.05 M spermidine was agitated using a low-speed vortex and then sonicated at 35 kHz. The 50 µL mixture of CLCuGeV and CLCuGeB DNA was added and then gently vortexed for 5 s while adding 100 µL of cold 1 M CaCl_2_ drop by drop. After 10 min of incubation at room temperature and centrifugation for 15 s at 16,000× *g*, the supernatant was removed. The pellet was then washed three times with cold absolute ethanol and the DNA-coated gold beads were transferred into a PVP solution at 0.05 mg/mL. The cartridges were assembled using Tetzel-type plastic tubes that had been sterilized by 2 or 3 washes with absolute ethanol and then dried using liquid nitrogen. The mixture containing the resuspended gold beads was evenly distributed with the Bio-Rad syringe kit into the central part of the Tetzel tube. After 2 min, beads were dried for 5 min using liquid nitrogen. The cartridges were then used immediately. The undersides of the cotyledons of 43 3-week-old okra plants were bombarded using a Helios Gene Gun System (Bio-Rad, San Francisco, CA, USA). 

Negative controls for PCR and qPCR tests consisted of 6 non-inoculated okra plants and 9 tomato plants agroinoculated with a mock preparation of the C58 (pMP90) strain of *A. tumefaciens* containing an empty pCAMBIA 2300 plasmid. 

All plants were grown in a containment chamber (14 h light at 26 °C and 10 h dark at 24 °C) and were watered with a solution containing 15:10:30 NPK fertilizer and micronutrients.

### 2.3. Transmission by the Vector Bemisia tabaci

Adult whiteflies belonging to the Q1 genotype of the Mediterranean species (MED) of the *B. tabaci* complex and originating from southern France (Tarascon) were used in the experiments.

**Figure 1:**
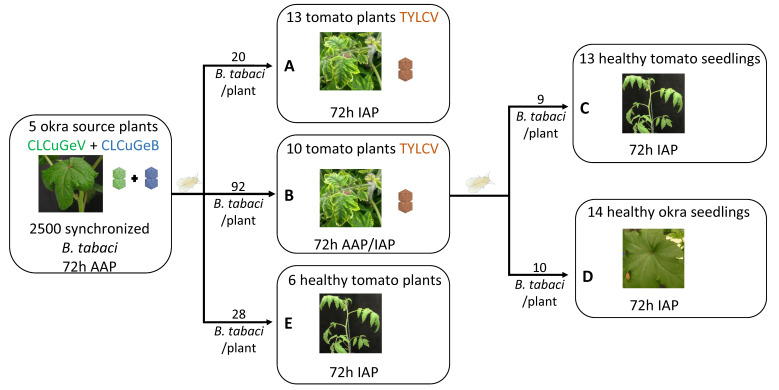
Schematic representation of the experimental design of vector transmission experiments using different batches of adult whiteflies (*Bemisia tabaci)*, (**A**–**E**). AAP: acquisition access period; IAP: inoculation access period.

The design of the transmission tests is described in Figure 1. Approximately 2500 *B. tabaci* adults collected from synchronized rearings on eggplants were given a 72 h acquisition access period (AAP) on 5 okra plants coinfected with CLCuGeV and CLCuGeB 3 months beforehand. In order to test for a potential transfer of CLCuGeB from CLCuGeV to TYLCV, viruliferous adult whiteflies were transferred for a 72 h inoculation access period (IAP) on 13 30-day-old TYLCV-infected tomato plants caged together (Figure 1A), to reach an average of 20 whiteflies/plant. A second batch with an average of 92 whiteflies/plant was transferred from the same okra source plants to another cage containing 10 TYLCV-infected tomato plants, for a 72 h AAP, allowing whiteflies to secondarily acquire TYLCV (Figure 1B). Viruliferous adult whiteflies were then transferred for a 72 h IAP into two cages containing healthy 15-day-old plants (Figure 1C, 13 tomato plants with an average of 9 whiteflies/plant; Figure 1D, 14 okra plants, with an average of 10 whiteflies/plant). The aim of these experiments was to test the transmission of TYLCV and CLCuGeB to tomato plants and of CLCuGeV and CLCuGeB to okra plants. The direct transmission of CLCuGeV and CLCuGeB from okra to tomato plants was tested by inoculating 6 15-day-old tomato plants with an average number of 28 viruliferous adult whiteflies/plant (Figure 1E).

During the AAP and IAP, plants were frequently shaken to ensure a homogenous distribution of insects. After the IAP, all insects of experiments A to E were aspirated and discarded. All the leaves present on the plants during the IAP were cut 15 days after the beginning of the IAP to eliminate the eggs laid by the whiteflies. The transmission success was assessed on recipient plants 30 days after the beginning of the IAP by symptom scoring, PCR, and qPCR.

### 2.4. Plant DNA Extraction

Tomato and okra recipient plants were sampled individually at 30 days postinoculation (dpi) by collecting five 4 mm diameter leaf disks from the youngest adult leaf of each plant. Okra source plants were collected in the same way at 90 days after biolistic inoculation. Total DNA was extracted using the protocol of Dellaporta et al. (1983) [54] modified as follows: leaf tissue was ground in 400 μL extraction buffer (100 mM Tris HCl pH 8.0, 50 mM EDTA, 500 mM NaCl, 10% SDS, 0.46% *w*/*v* Na_2_SO_3_, and 100 mg/mL RNase), incubated at 65 °C for 10 min, and centrifuged (16,000× *g* for 10 min). One volume of isopropanol was added to 300 μL of the supernatant, and nucleic acids were precipitated by centrifugation (16,000× *g* for 20 min); the pellet was washed with ethanol 70% and then resuspended in 250 μL of Milli-Q water. DNA extracts were used directly or stored at −20 °C.

### 2.5. PCR Detection and Quantitative PCR

To assess the infection status of plants after biolistic inoculation, agroinoculation, and vector transmission experiments, we performed PCRs with primer pairs CLCuGeV-84-F/CLCuGeV-507-R for CLCuGeV, CLCuGeB-282-F/CLCuGeB-699-R for CLCuGeB, and TY-451-F/TY-1029-R for TYLCV (Table 1). Each PCR was performed in a final volume of 25 µL containing 5 µL of 5× Green GoTaq Reaction Buffer (Promega, Madison, WI, USA), 1 µL of 5 mM of each dNTP, primer mix as mentioned in Table 1, 1.25 units of GoTaq DNA polymerase (Promega, USA), and 1 µL of total DNA extract. The PCRs were run under the following conditions: initial denaturation at 95 °C for 5 min followed by 30 cycles consisting each of a denaturation step at 95 °C (for 45 s for CLCuGeV and CLCuGeB, or 1 min for TYLCV), annealing and extension conditions as described in Table 1, and a 10 min final extension at 72 °C. Mock-inoculated tomato plants and non-inoculated okra plants were tested in parallel as negative controls.

The amount of viral or betasatellite targets in each plant was estimated by quantitative PCR (qPCR) using the LightCycler 480 SYBR Green II qPCR Master Mix (Roche, Mannheim, Germany). Primer pairs used are CLCuGeV-238-F/CLCuGeV-338-R for CLCuGeV, CLCuGeB-343-F/CLCuGeB-424-R for CLCuGeB, and TY-1431-F/TY-1576-R for TYLCV. The qPCRs were performed in a final volume of 10 μL containing the master mix 2×, 2 μL of total DNA extract diluted 1:20, and specific primers as described in Table 1. The 25S rRNA gene was used as a quality check of plant DNA extraction. Two technical replicates were performed for each DNA sample. The qPCRs were run in 384-well plates with the following cycling conditions: 95 °C for 10 min followed by 40 cycles consisting each of 10 s denaturation at 95 °C followed by annealing and elongation as described in Table 1 for each target. Mock-inoculated tomato plants and non-inoculated okra plants were used in parallel as negative controls.

The results were analyzed using LinRegPCR version 2021.1 [55], which calculates the starting concentration of the targeted amplicon (N_0_) expressed in fluorescence units. For each sample, the number of copies of each target was determined from the N_0_ values by linear interpolation from a log–log standard curve. This curve was obtained using 10-fold serial dilutions (ranging from 2 × 10^8^ to 2 × 10^1^ copies) of a PCR fragment containing the targeted amplicon obtained with primer pairs CLCuGeV-84-F/CLCuGeV-507-R for CLCuGeV, CLCuGeB-282-F/CLCuGeB-699-R for CLCuGeB, and TY-451-F/TY-1846-R for TYLCV (Table 1). The detection threshold was fixed at 756 targets, corresponding to the 1% upper quantile of the logistic distribution providing the best fit to the distribution of copy numbers for each of the 3 targets quantified in 32 extracts from healthy plants (okra: 14; tomato: 18).

Statistical analyses were performed using R Studio software version 4.3.1 (16 June 2023). DNA accumulations were compared between or within plants using log-transformed accumulation data. The log transformation was applied to normalize the data and stabilize the variance, ensuring that the assumptions of normality and homoscedasticity required for parametric tests were met. After performing Shapiro’s test that evidenced no significant departure from normality, we used Student’s *t*-test to compare the ratio of betasatellite to helper virus accumulation. Differences were considered statistically significant for *p*-values < 0.05.

## 3. Results

### 3.1. Success of Source Plant Inoculation

**Figure 2:**
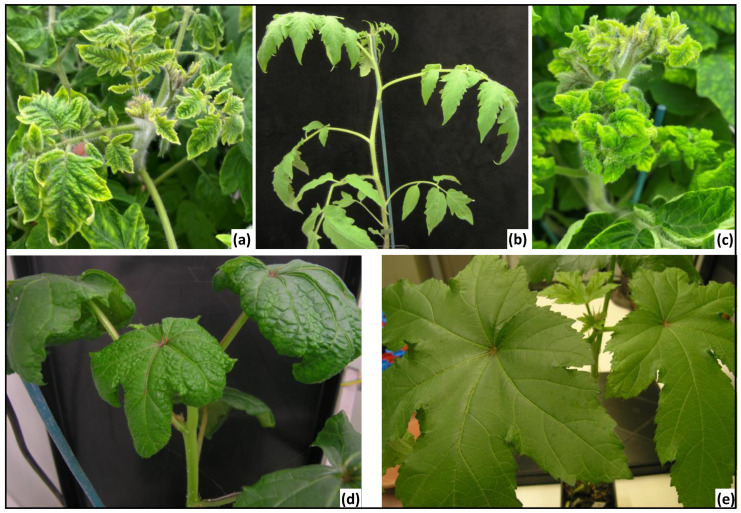
Symptoms on tomato and okra leaves at 30 dpi. (**a**) Symptomatic TYLCV-infected tomato plant. (**b**) Healthy tomato plant. (**c**) Symptomatic tomato plant infected with TYLCV + CLCuGeB. (**d**) Symptomatic okra plant infected with CLCuGeV + CLCuGeB. (**e**) Healthy okra plant.

At 30 dpi, 28/29 (96%) TYLCV-agroinoculated tomato plants displayed typical TYLCV symptoms (Figure 2a), such as moderate leaf curling, mild yellowing, and small leaves compared to the healthy plants (Figure 2b). The non-symptomatic plant was not analyzed. Out of the 28 symptomatic plants, 23 were randomly selected for the experiment. The efficiency of infection of okra plants was lower: only 5/43 (11%) of the okra plants inoculated by the biolistic method displayed symptoms such as vein thickening, curling, and stunting (Figure 2d) compared to the healthy plants (Figure 2e). PCR and qPCR tests confirmed the presence of TYLCV in the 23 tomato plants (Table S1, experiments A and B) and of both CLCuGeV and CLCuGeB in the 5 symptomatic okra plants (Table S2).

### 3.2. Transfer of a Betasatellite between Two Helper Viruses Infecting Different Hosts

The ability of the betasatellite (CLCuGeB) to be transferred from CLCuGeV to TYLCV was first tested in the situation where the recipient tomato plants were previously infected with TYLCV (experiment A). Thirty days after an IAP with an average number of 20 adult whiteflies/plant having acquired CLCuGeV + CLCuGeB, symptoms were scored on the 13 TYLCV-infected recipient tomato plants and the presence of TYLCV, CLCuGeV, and CLCuGeB was tested by PCR and qPCR; 6/13 plants (47%) tested positive for TYLCV alone and 7/13 plants (53%) for both TYLCV and CLCuGeB (Table 2). As expected, CLCuGeV was not detected in any of these 13 recipient tomato plants. The plants infected with TYLCV alone displayed typical slightly curved leaflets and light mosaic, as those shown in Figure 2a, while those infected with TYLCV and CLCuGeB displayed more severe symptoms including severe plant stunting and yellowing with pronounced cupping on new emerging leaves (Figure 2c). These results indicate that the betasatellite CLCuGeB can be successfully transmitted and assisted by TYLCV for replication and systemic infection in tomato plants, without the systemic infection of the initial helper virus (i.e., CLCuGeV).

### 3.3. Efficient Vector Transmission of Different Virus–Betasatellite Associations

The transfer between helper viruses was also tested in a condition where viruses and the betasatellite were acquired sequentially by the whiteflies. Whiteflies having first acquired CLCuGeB and CLCuGeV, and then TYLCV, were used to inoculate 13 healthy tomato plants (experiment C) and 14 healthy okra plants (experiment D). At 30 dpi, 12/14 okra plants (85%) displayed moderate to severe leaflet curling and yellowing. These plants tested positive for both CLCuGeV and CLCuGeB but negative for TYLCV (Table 2). Two plants were asymptomatic and tested negative for all targets (Table 2). Regarding tomato plants, all 13 recipient plants displayed symptoms at 30 dpi. While 3/13 plants (23%) tested positive for TYLCV alone and 1/13 plants (7%) tested positive for the three targets (TYLCV, CLCuGeV, and CLCuGeB), 9/13 plants (69%) tested positive for TYLCV and CLCuGeB but not for CLCuGeV (Table 2), indicating that the transfer between helper viruses can also occur when the virus and the satellites encounter each other in the whiteflies following different acquisition steps. The tomato plants infected with TYLCV alone displayed typical TYLCV symptoms (outward curling, yellowing, small leaves); the presence of CLCuGeB was often associated with more severe symptoms of stunting and leaf yellowing with pronounced cupping, whether CLCuGeV was detected or not. The detection of CLCuGeV in a tomato plant of experiment C was unexpected. Indeed, none of the six healthy tomato plants inoculated with whiteflies having acquired CLCuGeV and CLCuGeB on okra source plants (experiment E) tested positive for any target (Table 2), suggesting that this virus does not infect tomato plants, alone or with its betasatellite.

The 10 TYLCV-infected tomato plants of experiment B, on which an average number of 92 adult whiteflies/plant were released following a 72 h AAP on okra source plants infected with CLCuGeV and CLCuGeB, were tested 30 days post-AAP. Using such a high inoculum pressure, 6/10 plants (60%) tested positive for TYLCV, CLCuGeB, and CLCuGeV, whereas 3/10 plants (30%) tested positive for TYLCV and CLCuGeB, and 1/10 plants (10%) tested positive for TYLCV alone (Table 2). The analysis of virus accumulation in the six tri-infected tomato plants revealed that CLCuGeV accumulates 73 (range: 26–141) times less than TYLCV (Table S1, experiment B). Thus, CLCuGeV was detected in tomato plants, but only in coinfection with CLCuGeB and TYLCV, as also observed in one tomato plant from experiment C, inoculated with whiteflies having acquired the three components (Table 2). Taken together, the outcomes of experiments A, B, C, and E suggest that both TYLCV and CLCuGeB are required for the low-level infection of tomato plants with CLCuGeV.

### 3.4. Efficient Replication of the Betasatellite by Different Helper Viruses

**Figure 3:**
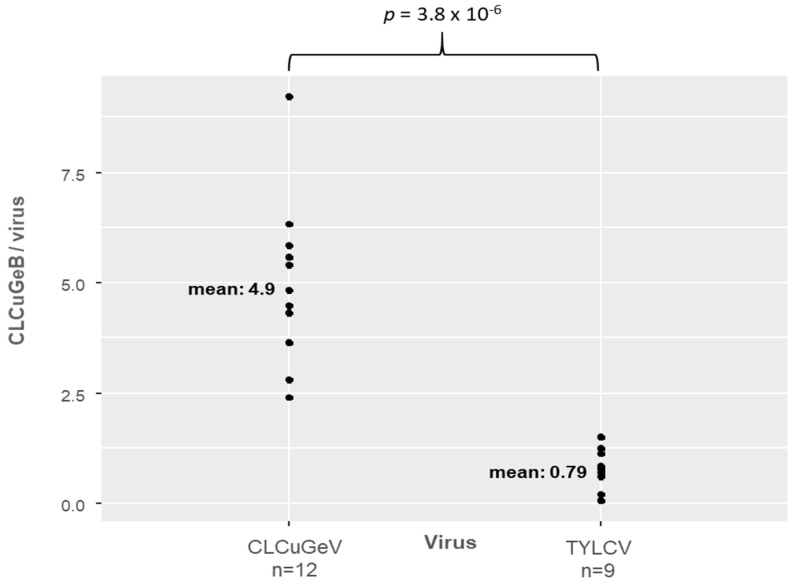
Accumulation ratio of betasatellite CLCuGeB to CLCuGeV in infected okra plants compared with CLCuGeB to TYLCV at 30 dpi in infected tomato plants. n: number of analyzed plants. *p*: *p*-value from a Student’s *t*-test for independent samples, indicating a significant difference between the means.

We have shown that whiteflies carrying two begomoviruses and a betasatellite can transmit different begomovirus–betasatellite associations to the respective host plants of each begomovirus and even CLCuGeV in tomato (which is considered as a non-host plant). We compared the accumulation of CLCuGeB relative to each helper virus in each host plant species (i.e., ratio of CLCuGeB to TYLCV in 9 tomato plants of experiment C and of CLCuGeB to CLCuGeV in 12 okra plants of experiment D). The mean ratio of betasatellite to helper virus is higher for CLCuGeV (4.93) than for TYLCV (0.79) (Figure 3 and Table S1) and this difference is statistically significant (*p* = 3.8 × 10^−6^). This means that there are 6 times more copies of betasatellite per copy of CLCuGeV in okra plants than per copy of TYLCV in tomato plants. A similar relation between accumulation ratios was also observed between tomato plants coinfected with TYLCV and CLCuGeB in experiment A (Table S1) and the five okra source plants coinfected with CLCuGeV and CLCuGeB (Table S2).

## 4. Discussion

The dissemination of betasatellites between helper begomoviruses and host plants in new environments is a matter of concern for the management of vegetable crops. In this study, we have tested the ability of the betasatellite CLCuGeB, initially acquired with CLCuGeV from okra plants, to form a new association with a different helper virus and host plant species. We also tested the spread of different virus–betasatellite associations to different plants species by whiteflies that have individually acquired all possible components.

We showed that an infectious clone of the betasatellite CLCuGeB, initially assisted by CLCuGeV in okra plants, can be transferred to TYLCV following vector transmission in TYLCV-infected tomato plants. A similar transfer was observed following transmission to healthy tomato plants with whiteflies having acquired CLCuGeV + CLCuGeB first, and then TYLCV. In both cases, the transmission of CLCuGeB or TYLCV + CLCuGeB was observed without the systemic detection of CLCuGeV. Our results suggest that betasatellites can invade new environments by disseminating with or without their original helper virus when they encounter a suitable helper virus in a suitable host plant or vector. This result is consistent with the reported detection of begomovirus–betasatellite associations involving helper viruses usually not associated with betasatellites: tomato yellow leaf curl Mali virus and CLCuGeB in tomato in Mali [28], TYLCV and CLCuGeB in Jordan [41] and Israel [42], or different combinations involving five distinct begomovirus species and seven betasatellites species in tomato fields in India [48]. In many cases, the described cognate helper viruses of these betasatellites were not detected, suggesting that the betasatellite may have been transmitted without its helper virus. Following multiple acquisition on different infected hosts, whiteflies can acquire different helper viruses and betasatellites and redistribute different combinations to different hosts, as shown in this study. Therefore, the dissemination of a betasatellite is not limited to the distribution and host plant of the cognate helper virus. To some extent, this situation is similar to the components of multipartite viruses that can reassort with components of other virus species and may cause new diseases [56].

Because the AAP on TYLCV-infected tomato plants (experiment B) lasted 72 h, one may imagine that, during this period, CLCuGeV and CLCuGeB acquired in okra were inoculated in tomato, replicated, and then reacquired by whiteflies. However, previous work monitoring TYLCV translocation in susceptible tomato plants inoculated during 24 or 48 h under high inoculum pressure (50–70 adult whiteflies per plant) showed that the viral DNA is not detected before 4 days in the plant [57,58]. Thus, we consider that most of (or all) the betasatellite particles inoculated into the healthy tomato or okra plants were acquired from the okra source plant and not from the intermediate tomato plants.

Our experimental design was based on the assumption that tomato is not a natural host of CLCuGeV. However, CLCuGeV was detectable 30 days after vector inoculation in 1/13 tomato plants among the 32 vector-inoculated plants of experiments A, C, and E, as well as in 6/10 TYLCV-infected tomato plants inoculated with a high number of adult whiteflies viruliferous for CLCuGeV and CLCuGeB (92 whiteflies/plant, experiment B). In all of these seven tomato plants, CLCuGeV was strictly associated with TYLCV and CLCuGeB, and its accumulation level was approximately 75 times less than that of TYLCV (Table S1). The ability of CLCuGeV to systemically infect tomato plants may be favored by the high inoculum pressure, as 6/10 plants could be infected using a mean number of 92 whiteflies/plant, versus 1/13 plants using a mean number of 9 whiteflies/plant. Similarly, vector transmission of African cassava mosaic virus to *Datura stramonium* plants was obtained only when more than 1000 insects were used per plant [59]. Although millions of viral genomes are inoculated in a plant by the vector [60], only a few genomes were found to initiate the systemic infection of the plant [61]. As the success of inoculation of the begomovirus cucurbit leaf crumple virus or TYLCV depends on the amount of virus in the inoculating whiteflies [62,63], a high inoculum pressure likely results in high numbers of inoculated genomes.

Moreover, coinfection with CLCuGeB and TYLCV seems to be a necessary condition for the infection of tomato plants by CLCuGeV. This is consistent with previous observations where CLCuGeV was detected in two tomato plants in Oman [64] and Saudi Arabia [65], always with TYLCV, betasatellites, and alphasatellites in tomato, or with tomato leaf curl Palampur virus (*Begomovirus solanumpalampurense*), alphasatellites, and betasatellites in muskmelon. Infection of tomato plants with CLCuGeV may be attributed to a possible complementation by TYLCV and CLCuGeB. TYLCV has been shown to complement or positively interact with other viruses [66,67,68]. Therefore, CLCuGeV infections in tomato plants might occur rarely, only when conditions for initiation and complementation with TYLCV and CLCuGeB are fulfilled. However, since CLCuGeV could infect some tomato plants systemically, we cannot exclude that it might have participated in the very first step of infection by replicating the betasatellite, even though CLCuGeV was not detected in newly developed leaves.

Coinfected plants pose specific epidemiological risks. As begomoviruses are highly recombinogenic [69,70], the coinfection of tomato plants with CLCuGeV, TYLCV, and CLCuGeB increases the opportunity to generate recombinant viral genomes, which could have different properties from the parental viruses. Coinfected plants may also contribute to the distribution of betasatellites with different begomoviruses in various host plants. For example, the weed *Sinapis arvensis* was detected as infected with five begomoviruses and a betasatellite in Jordan [44].

Previous results have shown that TYLCV replicates efficiently CLCuGeB in tomato plants [28,51] but no comparison has been performed with its original helper virus CLCuGeV. The relative accumulation data obtained in this study in different hosts suggest that CLCuGeB is more efficiently assisted by CLCuGeV in okra than by TYLCV in tomato.

In conclusion, the present study reports two processes by which a betasatellite can be transferred between helper viruses and/or hosts following vector transmission. The subsequent risk of dissemination of betasatellites in begomovirus-infected areas is expanded by the possible benefit for some viruses to associate with betasatellites in unusual host plants. The presence of betasatellites in the Middle East and eastern Mediterranean poses a significant threat to production in the western part of the Mediterranean Basin, which is already afflicted by numerous begomoviruses. The recent invasion of CLCuGeB in tomato production in Israel and Jordan is of particular concern, as commonly used TYLCV-resistant cultivars are overcome by the betasatellite [42,71]. It is worth mentioning that CLCuGeB was detected in 2011 in whiteflies from Israel [45]. However, the emergence of TYLCV + CLCuGeB epidemics was observed in tomato fields only in 2013 in Jordan and 2016 in Israel. This experience highlights the need for epidemiological surveillance of betasatellites, quarantine measures, and prompt reactions upon detection to moderate their effects.

## Figures and Tables

**Table 1:** Description of primers used for PCR and qPCR amplifications.

Targeted Viral Clone (Accession No.)	Primer Name ^a^	Primer Sequence (All in the 5′-to-3′ Direction)	Annealing Conditions	Extension Conditions	Primer Final Molarity
Tandem construct in pCAMBIA 2300 CLCuGeV (FN554531) CLCuGeB (FN55457)	M13-8436-F M13-8333-R	GTAAAACGACGGCCAG CAGGAAACAGCTATGAC	50 °C–50 s	69 °C 6 min for CLCuGeV 3 min for CLCuGeB	400 nM
CLCuGeV (FN554531)	CLCuGeV-84-F CLCuGeV-507-R	CTGTCCAATCAGAACGCGC TACATTCGGTACATCCTCGG	50 °C–45 s	72 °C–30 s	200 nM
CLCuGeB (FN55457)	CLCuGeB-282-F CLCuGeB-699-R	CACTTCGACTAACTCCTCCG TCGTATGAGCCTGTATGACG	54 °C–45 s	72 °C–30 s	200 nM
TYLCV (AM409201)	TY-451-F TY-1029-R	GCCCATGTAYCGRAAGCC GGRTTAGARGCATGMGTAC	57 °C–60 s	72 °C–30 s	200 nM
TYLCV (AM409201)	TY-451-F TY-1846-R	GCCCATGTAYCGRAAGCC TCATTGATGACGTAGACCC	54 °C–90 s	72 °C–60 s	200 nM
TYLCV (AM409201)	TY-1431-F TY-1576-R	AAACGCCATTCTCTGCC CACAAGATAGCCAAGAAGAAACC	60 °C–20 s	72 °C–20 s	300 nM
CLCuGeB (FN55457)	CLCuGeB-343-F CLCuGeB-424-R	AACCCATTCATTATTTC CGTTCATCATACCATA	52 °C–30 s	72 °C–20 s	300 nM
CLCuGeV (FN554531)	CLCuGeV-238-F CLCuGeV-338-R	TACCTTCAGGCTGTTCGAG GCTTCGACATAGTTAGTACGGCGG	62 °C–15 s	72 °C–17 s	600 nM
Tomato 25S rRNA gene (X13557)	25SRNA-1137-F 25SRNA-1297-R	AGAACTGGCGATGCGGGATG GTTGATTCGGCAGGTGAGTTGT	62 °C–20 s	72 °C–10 s	300 nM

^a^ Primer names include the name of the targeted genome, followed by the nucleotide position of the 5′ end, and ‘F’ for forward or ‘R’ for reverse sense.

**Table 2:** Infectivity of TYLCV, CLCuGeV (GeV), and CLCuGeB (GeB) in okra and tomato recipient plants following vector transmission.

Source Plant ^a^	Recipient Plant ^a^ (Experiment)	Average Number of Insects/ Plant	Number of Recipient Plants Analyzed ^b^	Infection Type							
				No Infection	TYLCV	GeV	GeB	TYLCV + GeB	GeV + GeB	TYLCV + GeV	TYLCV + GeV + GeB
Okra GeV + GeB	Tomato TYLCV (A)	20	13	0	6	0	0	7	0	0	0
Okra GeV + GeB	Tomato TYLCV (B)	92	10	0	1	0	0	3	0	0	6
Okra GeV + GeB then Tomato TYLCV	Tomato Healthy (C)	9	13	0	3	0	0	9	0	0	1
	Okra Healthy (D)	10	14	2	0	0	0	0	12	0	0
Okra GeV + GeB	Tomato Healthy (E)	28	6	6	0	0	0	0	0	0	0

**^a^** The status of source and recipient plants before vector inoculation is given under the name of the plant. ^b^ All analyzed plants had symptoms except those where no infection was detected.

## Data Availability

Data are contained within the article and Supplementary Materials.

## Supplementary Materials

The following supporting information can be downloaded at: https://www.mdpi.com/article/10.3390/v16091420/s1; Table S1: Infection status of okra and tomato recipient plants 30 days post vector inoculation, determined from PCR and qPCR results, and accumulation ratios. Table S2: Infection status of okra source plants 90 days post biolistic inoculation determined from PCR and qPCR results.
