# Supplementary material for: Demonstration of Insect Vector-Mediated Transfer of a Betasatellite between Two Helper Viruses
Source: Viruses. 2024 Sep 5;16(9):1420. doi: 10.3390/v16091420 (PMC11436227; doi:10.3390/v16091420)
Supplement: Supplementary file 1 [file viruses-16-01420-s001.zip › viruses-3129093-supplementary.pdf]

Table S1. Infection status of okra and tomato recipient plants 30 days post vector inoculation, determined from PCR and qPCR results, and accumulation ratios. Data are given for TYLCV-infected tomato plants of experiments A and B, tomato seedlings of experiment C and okra seedlings of experiment D.

Experiment A	PCR TYLCV	PCR CLCuGV	PCR CLCuGeB	qPCR TYLCV	qPCR CLCuGV	qPCR CLCuGeB	Infection status	CLCuGeB/ TYLCV
Tomato TYLCV - A 1	+	-	+	1.5E+06	ND	1.1E+06	TYLCV/CLCuGeB	0.72
Tomato TYLCV - A 2	+	-	-	7.4E+05	ND	ND	TYLCV	
Tomato TYLCV - A 3	+	-	+	2.4E+05	ND	3.5E+04	TYLCV/CLCuGeB	0.14
Tomato TYLCV - A 4	+	-	-	1.3E+05	ND	ND	TYLCV	
Tomato TYLCV - A 5	+	-	+	7.8E+05	ND	1.3E+04	TYLCV/CLCuGeB	0.02
Tomato TYLCV - A 6	+	-	-	5.9E+05	ND	ND	TYLCV	
Tomato TYLCV - A 7	+	-	+	1.0E+06	ND	9.1E+04	TYLCV/CLCuGeB	0.09
Tomato TYLCV - A 8	+	-	-	5.2E+05	ND	ND	TYLCV	
Tomato TYLCV - A 9	+	-	-	4.0E+05	ND	ND	TYLCV	
Tomato TYLCV - A 10	+	-	+	2.4E+06	ND	9.6E+05	TYLCV/CLCuGeB	0.39
Tomato TYLCV - A 11	+	-	+	9.1E+04	ND	4.6E+03	TYLCV/CLCuGeB	0.05
Tomato TYLCV - A 12	+	-	+	3.3E+05	ND	7.3E+04	TYLCV/CLCuGeB	0.22
Tomato TYLCV - A 13	+	-	-	4.3E+05	ND	ND	TYLCV	

+/-: a band of the expected size was obtained (+) or not (-) after PCR amplification from the plant sample

ND: number of copies below the detection threshold of 758 copies

following

Experiment B	PCR TYLCV	PCR CLCuGV	PCR CLCuGeB	qPCR TYLCV	qPCR CLCuGV	qPCR CLCuGeB	Infection status	TYLCV/ CLCuGeV
Tomato TYLCV - B 1	+	+	+	1.3E+06	4.8E+04	5.7E+05	TYLCV/CLCuGeV/CLCuGeB	26
Tomato TYLCV - B 2	+	-	+	1.2E+06	ND	5.3E+05	TYLCV/CLCuGeB	
Tomato TYLCV - B 3	+	-	+	8.4E+05	ND	3.9E+05	TYLCV/CLCuGeB	
Tomato TYLCV - B 4	+	+	+	1.3E+06	1.4E+04	5.7E+05	TYLCV/CLCuGeV/CLCuGeB	93
Tomato TYLCV - B 5	+	+	+	1.3E+06	1.2E+04	5.5E+05	TYLCV/CLCuGeV/CLCuGeB	113
Tomato TYLCV - B 6	+	+	+	2.9E+06	1.1E+05	2.1E+06	TYLCV/CLCuGeV/CLCuGeB	27
Tomato TYLCV - B 7	+	+	+	8.9E+05	6.3E+03	5.6E+05	TYLCV/CLCuGeV/CLCuGeB	141
Tomato TYLCV - B 8	+	-	+	7.4E+05	ND	3.2E+05	TYLCV/CLCuGeB	
Tomato TYLCV - B 9	+	+	+	8.1E+05	2.2E+04	2.6E+05	TYLCV/CLCuGeV/CLCuGeB	37
Tomato TYLCV - B 10	+	-	-	1.9E+05	ND	ND	TYLCV	

+/-: a band of the expected size was obtained (+) or not (-) after PCR amplification from the plant sample

ND: number of copies below the detection threshold of 758 copies

following

Experiment C	PCR TYLCV	PCR CLCuGV	PCR CLCuGeB	qPCR TYLCV	qPCR CLCuGV	qPCR CLCuGeB	Infection status	CLCuGeB/ TYLCV
Healthy tomato seedlings - C 1	+	-	+	5.1E+05	ND	3.2E+05	TYLCV/CLCuGeB	0.62
Healthy tomato seedlings - C 2	+	-	-	1.5E+05	ND	ND	TYLCV	
Healthy tomato seedlings - C 3	+	-	**+**	7.0E+05	ND	5.5E+05	TYLCV/CLCuGeB	0.78
Healthy tomato seedlings - C 4	+	-	+	9.5E+05	ND	1.4E+06	TYLCV/CLCuGeB	1.50
Healthy tomato seedlings - C 5	+	+	**+**	7.4E+05	6.8E+03	9.0E+05	TYLCV/CLCuGeV/CLCuGeB	
Healthy tomato seedlings - C 6	+	-	+	3.8E+05	ND	7.9E+04	TYLCV/CLCuGeB	0.21
Healthy tomato seedlings - C 7	+	-	-	7.4E+04	ND	ND	TYLCV	
Healthy tomato seedlings - C 8	+	-	+	1.1E+05	ND	9.6E+04	TYLCV/CLCuGeB	0.84
Healthy tomato seedlings - C 9	+	-	-	7.4E+04	ND	ND	TYLCV	
Healthy tomato seedlings - C 10	+	-	+	1.0E+05	ND	5.2E+03	TYLCV/CLCuGeB	0.05
Healthy tomato seedlings - C 11	+	-	+	8.6E+05	ND	9.8E+05	TYLCV/CLCuGeB	1.13
Healthy tomato seedlings - C 12	+	-	+	7.4E+05	ND	5.2E+05	TYLCV/CLCuGeB	0.70
Healthy tomato seedlings - C 13	+	-	+	5.7E+05	ND	7.1E+05	TYLCV/CLCuGeB	1.25

+/-: a band of the expected size was obtained (+) or not (-) after PCR amplification from the plant sample

ND: number of copies below the detection threshold of 758 copies

following

Experiment D	PCR TYLCV	PCR CLCuGV	PCR CLCuGeB	qPCR TYLCV	qPCR CLCuGV	qPCR CLCuGeB	Infection status	CLCuGeB/ CLCuGeV
Healthy okra seedlings - D 1	-	+	+	ND	1.8E+03	1.7E+04	CLCuGeV/CLCuGeB	9.23
Healthy okra seedlings - D 2	-	+	+	ND	2.2E+05	1.0E+06	CLCuGeV/CLCuGeB	4.49
Healthy okra seedlings - D 3	-	+	+	ND	4.5E+04	2.2E+05	CLCuGeV/CLCuGeB	4.83
Healthy okra seedlings - D 4	-	+	+	ND	8.1E+04	2.9E+05	CLCuGeV/CLCuGeB	3.63
Healthy okra seedlings - D 5	-	+	+	ND	4.6E+03	2.0E+04	CLCuGeV/CLCuGeB	4.30
Healthy okra seedlings - D 6	-	+	+	ND	2.7E+04	1.2E+05	CLCuGeV/CLCuGeB	4.32
Healthy okra seedlings - D 7	-	+	+	ND	1.4E+05	8.3E+05	CLCuGeV/CLCuGeB	5.85
Healthy okra seedlings - D 8	-	-	-	ND	ND	ND	no infection	
Healthy okra seedlings - D 9	-	-	-	ND	ND	ND	no infection	
Healthy okra seedlings - D 10	-	+	+	ND	1.8E+05	1.1E+06	CLCuGeV/CLCuGeB	6.33
Healthy okra seedlings - D 11	-	+	+	ND	8.8E+03	4.9E+04	CLCuGeV/CLCuGeB	5.58
Healthy okra seedlings - D 12	-	+	+	ND	1.4E+04	7.4E+04	CLCuGeV/CLCuGeB	5.42
Healthy okra seedlings - D 13	-	+	+	ND	8.3E+03	2.3E+04	CLCuGeV/CLCuGeB	2.80
Healthy okra seedlings - D 14	-	+	+	ND	4.2E+04	1.0E+05	CLCuGeV/CLCuGeB	2.39

+/-: a band of the expected size was obtained (+) or not (-) after PCR amplification from the plant sample
ND: number of copies below the detection threshold of 758 copies

Table S2. Infection status of okra source plants 90 days post biolistic inoculation determined from PCR and qPCR results. All plants showed stunted plant growth. tight leaves with yellowing on young leaves.

Source plant	PCR TYLCV	PCR CLCuGeV	PCR CLCuGeB	qPCR TYLCV	qPCR CLCuGeV	qPCR CLCuGeB	Infection status
Okra 2	nt	+	+	ND	6.2E+05	1.2E+06	CLCuGeV/CLCuGeB
Okra 7	nt	+	+	ND	1.1E+05	2.2E+05	CLCuGeV/CLCuGeB
Okra 11	nt	+	+	ND	7.1E+05	8.7E+05	CLCuGeV/CLCuGeB
Okra 24	nt	+	+	ND	1.6E+05	3.3E+05	CLCuGeV/CLCuGeB
Okra 43	nt	+	+	ND	1.5E+05	3.1E+05	CLCuGeV/CLCuGeB

nt: not tested
+: a band of the expected size was obtained after PCR amplification from the plant sample
ND: number of copies below the detection threshold of 758 copies
